# The Potential of HLA-G-Bearing Extracellular Vesicles as a Future Element in HLA-G Immune Biology

**DOI:** 10.3389/fimmu.2016.00173

**Published:** 2016-05-04

**Authors:** Vera Rebmann, Lisa König, Fabiola da Silva Nardi, Bettina Wagner, Luis Felipe Santos Manvailer, Peter A. Horn

**Affiliations:** ^1^Institute for Transfusion Medicine, University Hospital Essen, Essen, Germany; ^2^Department of Gynecology and Obstetrics, Essen, Germany; ^3^Laboratory of Immunogenetics and Histocompatibility (LIGH), Federal University of Paraná Genetics Department, Curitiba, Paraná, Brazil; ^4^CAPES Foundation, Ministry of Education of Brazil, Brasília, Federal District, Brazil

**Keywords:** extracellular vesicles, HLA-G, sHLA-G, LILRB1, LILRB2, KIR2DL4, exosome, HLA-G-bearing EV

## Abstract

The HLA-G molecule is a member of the non-classical HLA class I family. Its surface expression is physiologically restricted to the maternal–fetal interface and to immune privileged adult tissues. Despite the restricted tissue expression, HLA-G is detectable in body fluids as secreted soluble molecules. A unique feature of HLA-G is the structural diversity as surface expressed and as secreted molecules. Secreted HLA-G can be found in various body fluids either as free soluble HLA-G or as part of extracellular vesicles (EVs), which are composed of various antigens/ligands/receptors, bioactive lipids, cytokines, growth factors, and genetic information, such as mRNA and microRNA. Functionally, HLA-G and its secreted forms are considered to play a crucial role in the network of immune-regulatory tolerance mechanisms, preferentially interacting with the cognate inhibitory receptors LILRB1 and LILRB2. The HLA-G mediated tolerance is described in processes of pregnancy, inflammation, and cancer. However, almost all functional and clinical implications of HLA-G *in vivo* and *in vitro* have been established based on simple single ligand/receptor interactions at the cell surface, whereas HLA-G-bearing EVs were in minor research focus. Indeed, cytotrophoblast cells, mesenchymal stem cells, and cancer cells were recently described to secrete HLA-G-bearing EVs, displaying immunosuppressive effects and modulating the tumor microenvironment. However, numerous functional and clinical open questions persist. Here, we (i) introduce basic aspects of EVs biology, (ii) summarize the functional knowledge, clinical implications and open questions of HLA-G-bearing EVs, and (iii) discuss HLA-G-bearing EVs as a future element in HLA-G biology.

## Introduction

HLA-G is a non-classical HLA class I molecule. It is a potent suppressive molecule that impairs effector functions of immune cells belonging to the innate and adaptive immune system. Under physiological conditions, its surface expression is restricted to the maternal–fetal interface and to immune privileged adult tissues (1). However, secreted soluble forms of HLA-G are detectable in a variety of body fluids such as peripheral blood and amniotic fluids (2), malignant ascites (3, 4), pleural effusions (5), cerebrospinal fluid (6, 7), and sperm (8). Neo-ectopic or aberrant expression of HLA-G has frequently been related to malignancies (9–13), viral infections (14–19) including liver-related hepatitis B (16) and C (18) virus infections, autoimmune disorders (20–22), inflammatory diseases (23), complications (24, 25), and transplantation outcomes (26, 27).

A unique feature of HLA-G is that it exists in multiple structures, either expressed on the cell surface or in a secreted form. These different forms can mainly be attributed to alternative splicing of the primary transcript and differential association with β2-microglobulin (β2m). Four isoforms (HLA-G1, G2, G3, and G4) are membrane-expressed and three isoforms express either intron 4 (HLA-G5 and -G6) or intron 2 (HLA-G7) but lack the transmembrane and cytoplasmic domains, resulting in their secretion. With the exception of HLA-G3 (28), all HLA-G structures can create disulfide bounds between two unique cysteine residues at positions 42 (Cys42–Cys42 bonds) and 147 (Cys42–Cys147 bonds) (29, 30). The structures displaying the full-length extracellular domain (HLA-G1 and HLA-G5) are probably the most frequently detected. The structural diversity is further enhanced in that all membrane-expressed structures can also be shed from cell surface by metalloproteases (31) or can be secreted *via* extracellular vesicles (EVs) (32).

Regarding function, HLA-G and the soluble counterparts preferentially exert their immune modulating or suppressing functions by interaction with the two inhibitory receptors, leukocyte immunoglobulin-like receptor subfamily B member 1 (LILRB1) and LILRB2. LILRB1 is expressed on subpopulations of T-cells, B-cells, and Natural Killer (NK) cells. Monocytes/macrophages/dendritic cells (DC) express both receptors. These two receptors distinguish between β2m-associated and β2m-free HLA-G: LILRB1 interacts with HLA-G molecules associated to β2m, whereas LILRB2 specifically recognizes β2m-free HLA-G (33, 34). HLA-G dimers bind to LILRB with a higher affinity and avidity than monomers, resulting in more efficient LILRB-mediated signaling (35, 36). Additionally, HLA-G has been described to be the sole ligand for the killer immunoglobulin-like receptor 2DL4 (KIR2DL4), exhibiting both an activating and an inhibitory signaling domain. Moreover, soluble forms of HLA-G are able to trigger apoptosis in CD8^+^ T and NK cells (37) as well as in CD160-bearing endothelial cells (38).

Based on the functionality of receptors and their expression profile, membrane-expressed and soluble forms of HLA-G molecules are involved in immune regulation in pregnancy, inflammation, and cancer. Thus, HLA-G can be considered as an immune checkpoint molecule (39). However, most functional implications of HLA-G *in vivo* and *in vitro* have been deduced from the HLA-G1 and HLA-G5 structures and from a rather simple point of view on single ligand/receptor interaction. Interaction of target cells with HLA-G-bearing EVs has typically not been considered. Here, we (i) introduce basic aspects of EVs biology, (ii) summarize the current knowledge and open questions of HLA-G-bearing EVs, and (iii) discuss HLA-G-bearing EVs as a future element in the HLA-G biology.

## Basic Aspects of EV Biology

### Common Features of Extracellular Vesicles

Extracellular vesicles are phospholipid bilayer-enclosed vesicles, which are released by most cell types, including immune cells, tumor cells, stroma cells, trophoblast cells, and adult and embryonic stem cells (40). Depending on the cell of origin, state, and micro-environment, EVs are highly heterogeneous in size, membrane composition, and molecular content. According to biogenesis, EVs are specified as exosomes (70–150 nm), microvesicles (100–1000 nm), and apoptotic bodies (AB) (>500 nm). Exosomes correspond to intraluminal vesicles (ILVs), formed from inward budding of small-sized plasma membrane and enclosed in multi-vesicular bodies (MVB). Exosomes are released into extracellular space after fusion of MVB with the plasma membrane (41). In contrast, microvesicles (MV) are formed by outward budding and sission of the plasma membrane. AB are generated from plasma membrane blebs of cells undergoing apoptosis. Oncosomes, which are generated by the shedding of plasma membrane blebs of non-apoptotic cancer cells (42), and form an atypically large EV population (1,000–10,000 nm). Several proteins are currently used as markers for EVs, including tetraspanins, different heat shock proteins, adhesion molecules, cytoskeletal proteins, and members of endosomal sorting complexes required for transport of exosomes like TSG101 (43, 44). However, so far, no specific markers have been identified allowing for the identification of particular EV subpopulations (44).

Different cell types release differently assembled EV. Furthermore, it is tempting to speculate that even individual cells release different EV types. Importantly, the cell of origin controls the molecular composition and cargo (45–48). EVs harbor various types of antigens, cell surface-expressed receptors or ligands including classical and non-classical HLA-G (32, 49–53), bioactive lipids such as prostaglandins (54) and leukotrienes (55). Additionally, EVs can serve as transport cassettes or a disseminated storage pool of bioactive effector molecules, e.g., cytokines transcription factors, growth factors, oncogenic proteins, and genetic information such as mRNA, microRNA (56–59). Here, the lipid membrane of EVs protects their contents against enzyme degradation present in body fluids and thereby facilitate the transfer of their cargo over a short or long distance.

### Modes of Interaction between Extracellular Vesicles and Target Cells

The composition of EVs is responsible for the biodistribution, for the interaction of EVs to target cells or to extracellular matrix. Membrane fusion of EVs to target cells allows the transfer of bioactive molecules, including, e.g., CCR5 (60) and EGFRvIII (61), modifying the recipient cell phenotype. However, the direct fusion of EVs with the plasma membrane of effector cells requires a similar fluidity of the two fusing membranes. This can be achieved in an acidic micro-environment, which naturally occurs inside tumors (62–65) or at neutral pH in the presence of syncythin (66).

Besides membrane fusion, EVs can be internalized by different pathways including phagocytosis, clathrin- and caveolin-mediated endocytosis, or micropinocytosis (67). With the exception of the latter, the uptake and internalization of EVs are mostly receptor-mediated, e.g., *via* Hsp90 receptor or scavenger receptor CD36 (66). The expression of adhesion molecules on EVs probably facilitates the specific uptake of EVs, and their internalization by their cognate receptors being expressed on certain tissue or cell populations (68). The internalization of EVs results in the delivery and enrichment of bioactive molecules into the target cell’s endosomes. Hence, these molecules may be forwarded to other cell compartments, where they may contribute to an intracellular signaling mechanism.

### The Immunological Potential of Extracellular Vesicles

The communication and immune modulation by EVs take place among cells within same entity or between different types of cells. Various effector cells of the innate and adaptive immune system, including T cells and NK cells, antigen presenting cells (APCs), and mast cells have been reported to donate or to acquire ligand/receptor/genetic information *via* EVs. Due to the complex and often antagonistic composition, EVs can mediate gene expression modification, immune activating or immune suppression, introducing homeostasis or immune tolerance by the induction of T cell apoptosis, impairment of DC maturation, or the prevention of NK and T cell cytotoxicity (68–76). Furthermore, the molecular transfer of miRNA by EVs can alter the expression profile of the recipient cell (71). Tumor-derived EVs can stimulate immune suppression and tumor progression in different ways including the inhibition of tumor-specific T cell function and proliferation (77), the promotion of regulatory T cells subsets (78), and transfer of oncogenic receptors (61).

## The Current Status and Open Questions of HLA-G-Bearing Extracellular Vesicles

### HLA-G-Bearing EVs and Cancer

Without any doubt, the neo-ectopic expression of HLA-G molecules either on the surface of tumor cells or released as soluble forms can be considered a critical factor for cancer progression. Albeit high blood levels of soluble forms of HLA-G have concordantly been related to cancer, the prognostic relevance of soluble HLA-G in the blood has not always been established as an independent marker in terms of disease progression and survival (39). To date, the source of soluble HLA-G is known. In addition, it is not clarified whether HLA-G-bearing EVs or free soluble HLA-G (sHLA-G_free_) are produced by tumor cells and whether both subcomponents contribute to immune evasion of tumor cells.

Secreted HLA-G-expressing EVs (Table 1) have been detected for the first time in supernatants of a melanoma cell line, originated from a HLA-G-positive melanoma lesion (32). Both, the cell line cells and the secreted EVs express the full-length isoform HLA-G1. Up to now, it is not known whether HLA-G1-bearing EVs are functionally active to transduce inhibitory signals toward effector cells *via* the LILRB1/2 receptors, which may spread the tolerogenicity of HLA-G.

**Table 1:** **Source of HLA-G-bearing EVs with potential immunological and clinical relevance**.

Cell type	EVs source	Potential target cell response	Function/mechanism	Clinical relevance	Reference
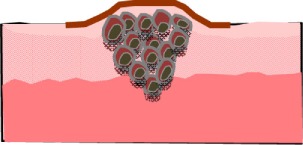	Melanoma	Tolerance-inducing effect of melanoma derived HLA-G-bearing EVs on immune cells	Potential induction of inhibitory signaling by HLA-G1-bearing EVs *via* LILRB1/2 receptors	Unknown clinical relevance	(32)
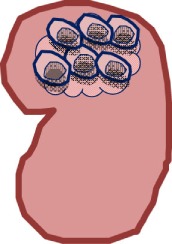	Kidney cancer	Inhibitory effect of HLA-G-bearing EVs on monocyte differentiation into mature DCs and reduced T cell proliferation	Inhibitory effect of HLA-G1-bearing EVs on monocyte differentiation and their maturation to DCs	Suppression of immune effector cells by HLA-G1-bearing EVs, leading to disease progression	(83)
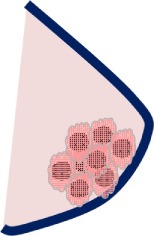	Breast cancer	Modulation of immune effector functions by circulating HLA-G-bearing EVs	Unknown function	Association of high circulating amounts of HLA-G-bearing EVs to disease progression	(84)

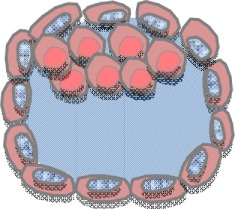	Trophoblast	Modulation of immune effector functions by cytotrophoblast-derived HLA-G5-bearing EVs	Unknown function	Unknown clinical relevance, but potential biomarker for pregnancy-related disorders	(87)

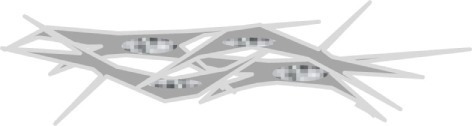	Mesenchymal stem/stromal cells (MSCs)	Induction of tolerance between graft and host immune cells by MSCs-derived EVs	Immunomodulation by synergistic additive effect of HLA-G, IL-10, and TGFβ	Potential therapeutic option for patients with therapy–refractory GvHD using MSC-derived HLA-G-bearing EVs	(91)

The first *in vivo* existence of HLA-G-bearing EVs was reported for ascites and pleural exudates derived from cancer patients (53). The EV fractions, however, contain ubiquitinated HLA-G molecules with atypically high HLA-G molecular sizes ranging from 50 to 75 kD. Generally, ubiquitination is a frequent posttranslational protein modification, by which proteins are targeted to protein degradation or directed to other cellular locations (79, 80). Interestingly, EVs contain many polyubiquitinated proteins, which are not integrated into their membrane (81). Thus, the presence of secreted HLA-G5 or HLA-G6 cannot be excluded.

Very recently, we established the prognostic relevance of HLA-G-bearing EVs for neoadjuvant chemotherapy-treated (NACT) breast cancer patients for the first time (82). Both, the total amount of HLA-G_tot_ and the amount of sHLA-G_free_ were significantly increased in breast cancer patients. Before NACT, sHLA-G_free_ levels are exclusively related to estrogen receptor expression, whereas high amounts of HLA-G in EVs (sHLA-G_EV_) enriched from peripheral blood samples are associated with the existence of circulating stem cell-like tumor cells. Strikingly, despite high amounts of sHLA-G_tot_, its prognostic relevance could not be substantiated. However, different impacts on prognosis have been shown for the two subcomponents sHLA-G_EV_ and sHLA-G_free_: high sHLA-G_EV_ levels are associated with disease progression, whereas high sHLA-G_free_ levels are related to an improved clinical outcome. This suggests that some of the sHLA-G_free_ molecules are impaired regarding LILRB1 recognition, and thereby are not qualified to exert inhibitory functions, as already demonstrated in rheumatoid arthritis patients (83). In conclusion, this study exemplifies the importance of stratifying soluble forms of HLA-G into free and EVs-bound molecules, as these two subcomponents can display diametrically opposed prognostic impact on disease progression likely due to the differential power of these compounds to contribute to an immune escape of tumor cells.

Further underlining the functional relevance of HLA-G-bearing EVs in cancer, a recent study demonstrated that (i) EVs released by renal cancer stem cells carry HLA-G with a HLA-G1 typical molecular weight, (ii) these HLA-G-bearing EVs impair the differentiation of monocytes to mature DCs, and (iii) the presence of these DCs reduces the T cell proliferation. Thus, HLA-G-bearing EVs mediate inhibitory effects on monocyte differentiation and their maturation to DCs (84).

### HLA-G-Bearing EVs and Pregnancy

At the maternal–fetal interface, HLA-G and its soluble forms are expressed on both sides, on extravillous trophoblast cells lining the placenta and on tolerance-inducing DCs (DC-10) being enriched in the first trimester decidua (25, 85). Thus, HLA-G is thought to orchestrate the cross talk among embryo trophoblasts, decidual leukocytes, and stromal cells allowing the trophoblast invasiveness, decidual cell differentiation, vascular remodeling, and the reprograming of local maternal immune responses (86). Whether HLA-G-bearing EVs represent an additional instrument to mediate communication of these cells is currently unclear. Interestingly, both first trimester and term placentas have been reported to secrete HLA-G5 isoforms *via* EVs (87). In agreement with the reported immunolocalization of HLA-G (88) cytotrophoblast cells, but not differentiated syncytiotroblasts, are producing HLA-G5-positive exosomes. The observation of the presence of HLA-G5 in EVs raises the issue whether HLA-G5 is associated with the luminal or with the extravesicular EV side. As secreted molecules, the association of HLA-G5 with extravesicular EV sides would require a binding partner it can associate with. Alternatively, the association with the luminal side would require the transit of HLA-G into the cytoplasma after biosynthesis. Independently of the immunogenicity of EVs and of the secretion pathway directing HLA-G5 toward EVs, it is clear that HLA-G5 isoforms being inside of EVs are hidden, which provokes questions about the function of HLA-G5 in cytotrophoblast-derived EVs.

### HLA-G-Bearing EVs and Mesenchymal Stem/Stromal Cells

Similar to trophoblast cells, mesenchymal stem/stromal cells (MSCs) express surface-expressed and soluble forms of HLA-G, which are involved in the suppression of T and NK cell functions (89). Besides HLA-G, MSCs exert the immune regulatory and modulatory activities through a variety of soluble mediators such as IL10, TGFβ, either as free soluble molecules or *via* immunological active EVs (90). The latter have been suggested to mediate synergistical effects of these molecules. In view of this, MSC-derived EVs, containing huge amounts of HLA-G, IL-10 and TGFβ, were used to treat a patient suffering from severe and therapy–refractory cutaneous and intestinal GvHD grade IV (91). After serial application rounds of MSC-EVs, a substantial improvement of the clinical GvHD symptoms has been achieved without any side-effects. Simultaneously, the allogeneic cytokine responsiveness of the patient’s peripheral mononuclear blood cells was substantially reduced. Although a direct impact of HLA-G on the immune suppression has not been demonstrated, this study represents the first treatment in humans, in which HLA-G with the immune modulatory function of MSC-derived EVs has been applied. Thus, it triggered significant interest in applying EVs-based therapeutics in clinical trials (92).

### New Perspectives of HLA-G-Bearing Extracellular Vesicles

Currently, the known functions of HLA-G are restricted to receptors expressed on the surface of effector cells of the innate and adaptive immune system. In this way, HLA-G inhibits the cytolytic function of NK cells (93, 94), the antigen-specific cytolytic function of cytotoxic T lymphocytes (CTL) (95) and γ/δ T cells (96), the allogeneic proliferative response (95), and proliferation of CD4+ T cells (97). HLA-G also impairs the maturation and function of DC (98, 99). Furthermore, HLA-G is related to regulatory cells including regulatory T cells (89, 100–102), regulatory DC (103), and myeloid-derived suppressor cells (104). Due to the differential composition of EVs, other compounds of the EVs may potentiate or abrogate the functional power of HLA-G. Additionally, EVs harboring HLA-G may allow the interaction with target cells lacking the surface expression of HLA-G specific receptors.

Membrane fusion of EVs to target cells can represent a possible mode of how HLA-G can be transferred to target cells. In this context, it is noteworthy that a cellular translocation of HLA-G from APCs to activated T cells (102) and from tumor cells to T/NK cells has been reported (105, 106). The acquisition of HLA-G reverses the function of T and NK cells to regulatory cells impairing allo-immune responsiveness. Such a spatiotemporal mechanism is suggested to be an instrument for “emergency” immune suppression used by HLA-G-expressing tissues to protect themselves against aggressive immune intervention (102). It is tempting to speculate that EVs mediate a transfer of HLA-G to effector cells, which would abrogate at least the regional mode of action.

Independent of the pathway, internalization of HLA-G-bearing EVs provides the opportunity for HLA-G to participate in yet unknown intracellular pathways. Interestingly, both soluble HLA-G5 and shed HLA-G1 have been reported to be bound by the transiently expressed KIR2DL4 receptor and to be endocytosed into early endosomes of NK cells (107–109). This leads to the activation of a nuclear factor-κB-pathway and finally to the transcription of pro-inflammatory and proangiogenic factors. Thus, the sustained endosomal signaling by KIR2DL4/HLA-G may allow NK cell activation despite a potential dominant inhibitory receptor–ligand interaction at cell surface. In context with the secretion of HLA-G by fetal trophoblast cells, this NK cell-mediated mechanism has been discussed to be operative in the promotion of vascularization in maternal decidua during early pregnancy (107–109). Here the question arises, whether this KIR2DL4–HLA-G pathway becomes operative when fetal trophoblast cells secrete HLA-G-bearing EVs or whether other yet unknown receptors can mediate intercellular signaling. The investigation of molecular signature molecules on HLA-G-bearing EVs may help to provide an insight into the functional consequence and the intracellular signaling pathway after internalization (69).

Regarding the role of HLA-G in diagnosis, prognosis, and treatment, the cell-specific signature of HLA-G-bearing EVs may not only provide information about the potential target cells and about its potential interplay of the cognate receptor/ligand on target cells but also about the cells producing these EVs (110). In that way, the identification of the cellular source on HLA-G-bearing EVs, such as the detection of the tumor marker HER-2/neu, may offer unforeseen diagnostic opportunities to monitor the systemic health status/disease status and disease activity/progression.

## Conclusion

It is well established that tumor cells, cytotrophoblast cells, and MSCs secret HLA-G-bearing EVs in addition to non-vesicular soluble HLA-G. All of these cell types are highly capable of promoting immune tolerance and tissue remodeling. Mechanisms and functional consequences of HLA-G-bearing EVs and their specific contribution to the biology of these cells have yet to be determined. So far, the classical concept of HLA-G function is based on the interaction of HLA-G with receptors being expressed on the cell surface membrane. EVs, however, may serve as a ticket for HLA-G to interact directly with cells or to enter into the inside of cells. The internalization of HLA-G may introduce new pathways or yet unknown cognate receptors, by which HLA-G contributes to intracellular communication. In that way, HLA-G-bearing EVs are likely to represent an important element in the biology of HLA-G.

## Author Contributions

VR: concept and design, drafting of manuscript. VR and PH: critical revision of the manuscript for important intellectual points, supervision. LK, FN, BW, LM, VR, and PH: drafting of manuscript.

## Conflict of Interest Statement

The authors declare that the research was conducted in the absence of any commercial or financial relationships that could be construed as a potential conflict of interest.
